# Supplementary material for: Naïve CD8 T cell IFNγ responses to a vacuolar antigen are regulated by an inflammasome-independent NLRP3 pathway and Toxoplasma gondii ROP5
Source: PLoS Pathog. 2020 Aug 27;16(8):e1008327. doi: 10.1371/journal.ppat.1008327 (PMC7480859; doi:10.1371/journal.ppat.1008327)

(A)

```
                              Alternative start site              Signal peptide cleavage site
                                                     ↓                     ↓
CAST       MNKDRPCRLNSRHAEDFSLVMSCQQRLAVVQGSLFCTMKMGRHVVILACFAALASTVAHGLFRSSSYWNDPWPETGEDWA
GUY-DOS    ................................................................P...............
P89        ................................................................P...............
RUB        ................................................................P...............
TgCATBR5   ................................................................P...............
MAS        ..........................................................................A.....
GUY-KOE    ..........................................................................A.....
GUY-MAT    ..........................................................................A.....
VAND       ..........................................................................A.....
COUGAR     .............................................................Y..P...............
GPHT       .............................................................Y..P...............
BOF        .............................................................Y..P...............
GT1        .............................................................Y..P...............
ME49       .............................................................Y..P...............
VEG        .............................................................Y..P...............
CASTELLS   .............................................................Y..P.........T.....

                          Kb 96-103 epitope
CAST       EVVAQVKTTSERLYESVLAFRRLTTSATKRYSAYKKEKQLRPEALQLPHMWHVRNEIWRITDDLRNAKDQLRLLIRKQKA
GUY-DOS    ................................................................................
P89        ................................................................................
RUB        ................................................................................
TgCATBR5   ................................................................................
MAS        ................................................................................
GUY-KOE    ................................................................................
GUY-MAT    ................................................................................
VAND       ................................................................................
COUGAR     ............................V...................................................
GPHT       ............................V...................................................
BOF        ............................V...................................................
GT1        ............................V...................................................
ME49       ............................V...................................................
VEG        ............................V...................................................
CASTELLS   ............................V...................................................

CAST       LISILEGMRDKGDLKHWDDTTNICRKASGELREAEELVEREEEEYNIEAE
GUY-DOS    ........................................K.......V.
P89        ........................................K.......V.
RUB        ........................................K.......V.
TgCATBR5   ........................................K.......V.
MAS        .......L.G...R...N...S..........................V.
GUY-KOE    .......L.G...R...N...S..........................V.
GUY-MAT    .......L.G...R...N...S..........................V.
VAND       .......L.G...R...N...S..........................V.
COUGAR     .........G...R...N...S...............I..........V.
GPHT       .........G...R...N...S..........................V.
BOF        .........G...R...N...S..........................V.
GT1        .........G...R...N...S..........................V.
ME49       .........G...R...N...S..........................V.
VEG        .........G...R...N...S..........................V.
CASTELLS   ......RM.GN......N...S..........................V.
```

(B)

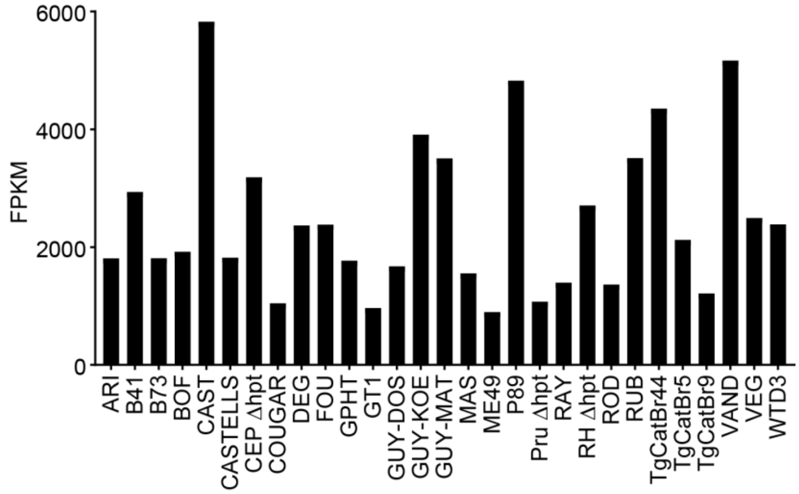

Supplement: S1 Fig — (A) Multiple protein alignment of TGD057 (215980) encoded by various T. gondii strains; the 96–103 MHC 1 Kb T57 T cell epitope is highlighted. Dots represent amino acid conservation with TGD057 from the CAST strain. The predicted signal peptide cleavage site and an alternative translational start site [58] is indicated with an arrow. (B) TGD057 gene expression (TG_215980) for 29 parasite strains following 20–22 hours post-infection in BMDMs (C57BL/6) is plotted from data previously reported [147]; expression values are in fragments per kilobase of exon model, per million mapped reads (FPKM). (PDF) [file ppat.1008327.s001.pdf]
